# Chemical sphincterotomy in posthemorrhoidectomy pain relief: a meta-analysis

**DOI:** 10.1186/s12893-023-02025-3

**Published:** 2023-05-09

**Authors:** Yifan Cheng, Misha Mao, Yaqian Shang, Chaomei Ying, Linnan Guo, Yong Lu

**Affiliations:** 1 Department of Gastrointestinal Surgery, Taizhou Hospital of Zhejiang Province Affiliated to Wenzhou Medical University, No. 150 Ximen Street, Linhai, Taizhou, 318000 Zhejiang China; 2 Department of Surgical Oncology, Sir Run Run Shaw Hospital, Zhejiang University, Hangzhou, Zhejiang China

**Keywords:** Chemical sphincterotomy, Haemorrhoidectomy, Calcium channel blockers, Glyceryl trinitrate, Botulinum toxin, Postoperative pain

## Abstract

**Purpose:**

This study aims to evaluate the pain relief function of chemical sphincterotomy in patients undergoing haemorrhoid surgery and compare, through a meta-analysis, the different drugs used to treat this condition.

**Methods:**

We conducted a search in databases including PubMed, EMBASE and Web of Science. The methodological quality was evaluated using the Revised Cochrane risk-of-bias tool for randomized trials (ROB2). The pain score was assessed using a visual analogue scale (VAS) on day 1, day 2, and day 7, and a meta-analysis was conducted based on the use of random effects models. In addition, the subgroup analysis was evaluated based on the kind of experimental drugs. Heterogeneity and publication bias were assessed.

**Results:**

Fourteen studies with a total of 681 patients were included in this meta-analysis, and all studies were randomized controlled trials RCTs. Chemical sphincterotomy showed better pain relief function than placebo on day 1 (SMD: 1.16, 95% CI 0.52 to 1.80), day 2 (SMD: 2.12, 95% CI 1.37 to 2.87) and day 7 (SMD: 1.97, 95% CI 1.17 to 2.77) after surgery. In the subgroup meta-analysis, we found that different drugs for chemical sphincterotomy provided different pain relief.

**Conclusion:**

Chemical sphincterotomy effectively relieves pain after haemorrhoidectomy, and calcium channel blockers have the best effect.

## Introduction

Haemorrhoid is one of the most common anal diseases in the world, and it is estimated that the lifetime risk of developing haemorrhoids in the general population may be as high as 75% [1]. Surgical treatment remains the primary modality indicated for high-grade haemorrhoids [2]. Postoperative pain, which generally results from a spasm of the internal anal sphincter, causes many issues for patients and is the main problem that decreases the postoperative satisfaction of patients [3]. To relieve the postoperative pain caused by spasm of the internal anal sphincter, patients often try to have the internal anal sphincter damaged, including through an internal sphincterotomy [4] or a chemical sphincterotomy through some drugs [5]. Internal sphincterotomy destroys the normal tissue of patients and may cause extra damage to them, which could induce faecal incontinence [6]. On the other hand, chemical sphincterotomy, which can reduce internal anal sphincter spasm, is safer [3, 7]. The drugs that are used for chemical sphincterotomy include calcium channel blocker (CCB), glyceryl trinitrate (GTN), and botulinum toxin (BTX). The function of chemical sphincterotomy in anal fissures is effective based on the latest research [8–10]. Some meta-analysis studies have demonstrated the effect of CCB [11] and GTN [12, 13] on the pain relief function of patients who underwent haemorrhoidectomy. However, few studies have studied chemical sphincterotomy by combining all the types of drugs for pain relief after haemorrhoidectomy. To elucidate whether chemical sphincterotomy can decrease the pain of patients after haemorrhoidectomy, we conducted this meta-analysis.

## Method

This meta-analysis is reported in accordance with the Preferred Reporting Items of the Systematic Review and Meta-Analysis (PRISMA) statement and is registered in the International Prospective Register of Systematic Reviews (PROSPERO, No. CRD42022357493).

### Search strategy

A comprehensive search of published studies was performed in PubMed, Embase and Web of Science. We combined the text word ("haemorrhoids" or "haemorrhoid") with (“calcium channel blocker” or “diltiazem” or “nifedipine”) OR (“glyceryl trinitrate” or "nitroglycerin" or “GTN”) OR ("botulinum toxin" or "BTX") OR ("chemical sphincterotomy"). No language restriction was applied. We did not perform any manual searches, and we did not contact the authors for unpublished relevant data.

### Eligibility criteria

Study selection was performed based on predefined Participants, Intervention, Comparators, Outcomes, Study design (PICOS) criteria.

#### Participants

Patients underwent hemorrhoidectomy, regardless of kind of surgery, were included.

#### Interventions

Any type of chemical sphincterotomy (including CCB, GTN and BTX injection) used as an intervention to release the pain after hemorrhoidectomy (regardless of the number and duration of the treatment) was included.

#### Comparators

Trials that compared chemical sphincterotomy versus placebo or other treatment for pain relief (including lidocaine or herbal ointment) were included. Studies comparing the efficacy of different kind of chemical sphincterotomy were excluded.

#### Outcomes

VAS score was used as the primary outcomes. The studies should report VAS score at least one of the following days after surgery: day1, day2 or day7 after surgery with standard deviation (SD).

#### Study design

Only randomized controlled trials (RCTs) were included. Dissertations, theses, guidelines, conference abstracts and narrative reviews were excluded.

Studies not meeting the criteria, studies without data for retrieval and duplicate publications were excluded. When two papers reported the same study, the publication that was more informative was selected.

### Data extraction

Two researchers (CYF and MMY) independently extracted data from the included studies by scrutinizing the full text and determining the methodological quality of all eligible studies. Disagreements were resolved by discussion or consensus or with a third reviewer (LY). The following information was collected from the eligible articles: authors, year of publication, location, number of patients with or without chemical sphincterotomy, kind of experimental drug use, patient age, sex, operation approach, and VAS score on days 1, 2, and 7 after surgery.

### Quality assessment

Three researchers (CYF, MMY and LY) used the ROB2 independently to assess the quality of RCTs [14]. Bias was assessed as a judgment (high, low, or some concerns) for elements from five domains: (1) randomization process; (2) deviations from intended interventions; (3) missing outcome data; (4) measurement of the outcome; and (5) selection of the reported result.

### Statistical analysis

The mean VAS score and SD of each study were collected and calculated using a random-effects model if the heterogeneity was considerable, and a fixed-effects model was performed otherwise. Heterogeneity analysis was performed by calculating the I^2^ index. We assessed the possibility of publication bias by Egger’s test. All statistical analyses were carried out using R version 4.2.0 software.

## Result

### Literature search

**Fig.1:**
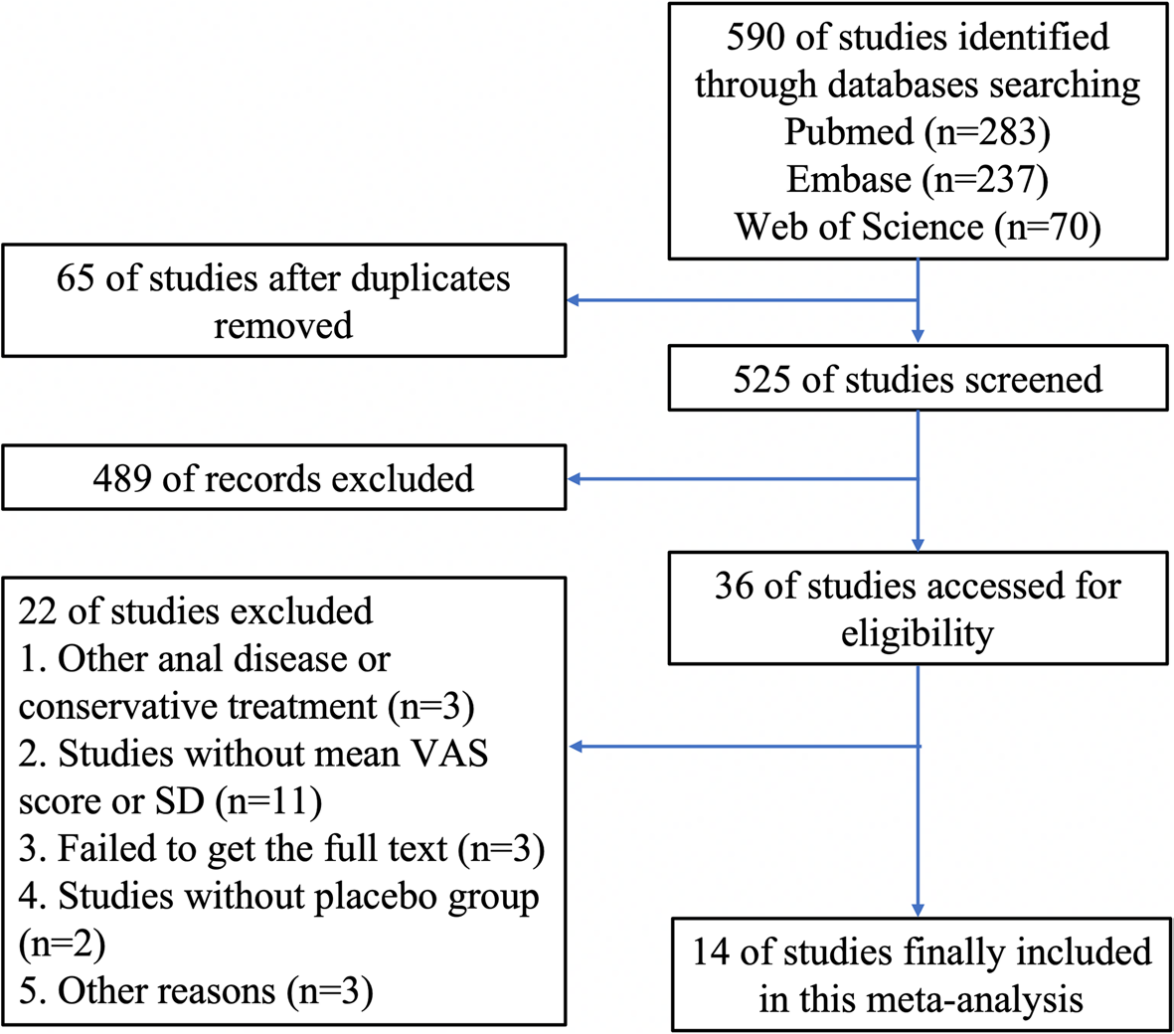
Flow chart showing the selection process for the included studies

Figure 1 shows the search process, which yielded a total of 590 citations using the search strategy. After excluding 65 duplicate and 525 irrelevant articles based on the abstracts or titles, we finally included 36 citations for detailed evaluation. After full-text reading, 14 studies matched our inclusion criteria and were included in our meta-analysis.

### Study characteristics

The characteristics of the 14 selected studies are presented in Table 1. These studies provide data on the VAS score of patients on different days after haemorrhoidectomy. All 14 studies were RCTs, and all of them were published after 2000. The experimental drug used in 5 of the studies was CCB ointment [15–19], in 7 of the studies, it was GTN ointment [20–26], and only 2 studies used BTX injection [27, 28], the administration was not same in different studies, CCB and GTN were applied to the perianal area at different frequencies daily after surgery, while BTX was injected into the intersphincteric space immediately after excision before closing the wound [27, 28]. All studies chose placebo drug in control group except 1 study [22] which used 2.5% lidocaine instead, all studies used the VAS score to evaluate the pain of patients. In the selection of operation, 7 studies included patients who underwent Milligan-Morgan haemorrhoidectomy [15, 17, 18, 20, 24, 26, 28], 4 studies chose Ferguson haemorrhoidectomy [19, 21, 23, 27], 1 study chose stapled haemorrhoidopexy [22], and 2 studies did not mention the surgery technique [16, 25].

**Table 1:** Baseline characteristics of the included studies

Studies	Year	Country	Study Design	Age (mean ± SD)		Sex (man: woman, n)		Operation	Primary Outcome	Specific Interventions
				**Control**	**Observation**	**Control**	**Observation**			
**Sunandan Yadav**	2018	India	RCT	50.40 ± 13.78	51.06 ± 12.92	23:7	22:8	Milligan-Morgan	VAS in 6 h and day1,2,7	OG: 2% diltiazem ointment CG: placebo Admn: 3 times daily × 1 week
**Ralph Silverman**	2005	USA	RCT	45.00 ± 5.00	44.00 ± 16.00	5:4	2:7	NM	VAS in day1-7	OG: 2% diltiazem ointment CG: placebo Admn: 3 times daily × 1 week
**H. A. Amoli**	2009	Iran	RCT	52.12 ± 12.99	54.13 ± 19.22	13:4	14:2	Milligan-Morgan	VAS in day1-7	OG: 2% diltiazem ointment CG: placebo Admn: 3 times daily × 1 week
**Sunil Suchdev**	2014	Pakistan	RCT	39.85 ± 14.91	37.73 ± 14.90	34:6	31:9	Milligan-Morgan	VAS in day2	OG: 2% diltiazem ointment CG: placebo Admn: NM
**U. Rodríguez-Wong**	2016	Mexico	RCT	44.77	46.59	6:11	9:8	Ferguson	VAS in day1-3	OG: 2% diltiazem ointment CG: placebo Admn: 3 times daily × 3 days
**Sepideh Vahabi**	2019	Iran	RCT	NM	NM	NM	NM	Milligan-Morgan	VAS in 6,12,18 and 24 h	OG: 0.2% GTN ointment CG: placebo Admn: 3 times daily × 1 week
**Harry J. Wasvary**	2001	USA	RCT	49	54	12:8	8:11	Ferguson	VAS in day1-7	OG: 0.2% GTN ointment CG: placebo Admn: 3 times daily × 1 week
**Francesco Saverio Mari**	2013	Italy	RCT	48.3 ± 8.6	48.7 ± 9	13:7	15:6	Stapled Hemorrhoidopexy	VAS in day1,2,7,14	OG: 0.4% GTN ointment CG: 2.5% lidocaine Admn: twice daily × 2 weeks
**Hasan Karanlik**	2009	Turkey	RCT	36.6 ± 10.4	34.4 ± 10.8	16:14	15:15	Ferguson	VAS in day1,3,7	OG: 0.2% GTN ointment CG: placebo Admn: twice daily × 2 weeks
**G. Di Vita**	2004	Italy	RCT	40.6 ± 18	35 ± 20	8:7	9:6	Milligan-Morgan	VAS in day1-7	OG: 0.2% GTN ointment CG: placebo Admn: 3 times daily × 2 weeks
**Do Yeon Hwang**	2003	Korea	RCT	NM	NM	NM	NM	NM	VAS in day1-3	OG: 0.2% GTN ointment CG: placebo Admn: 3 times daily × 3 weeks
**Rosalia Patti**	2005	Italy	RCT	36 ± 18	33 ± 15	8:7	9:6	Milligan-Morgan	VAS in day1,3,7	OG: 0.2% GTN ointment CG: placebo Admn: 3 times daily × 15 days
**Siripong Sirikurnpiboon**	2020	Thailand	RCT	42.23 ± 12.78	41.21 ± 13.94	23:20	21:18	Ferguson	VAS in 12 and 24 h	OG: 30 units of BTX CG: placebo Admn: Injection once after surgery
**B. Singh**	2009	UK	RCT	52.5 ± 11.4	52.9 ± 9.2	13:4	11:4	Milligan-Morgan	VAS in day1-14	OG: 150 units of BTX CG: placebo Admn: Injection once after surgery

*NM* Not Mentioned, *OG* Observation Group, *CG* Control Group, *Admn* Administration

### Risk of bias

**Fig. 2:**
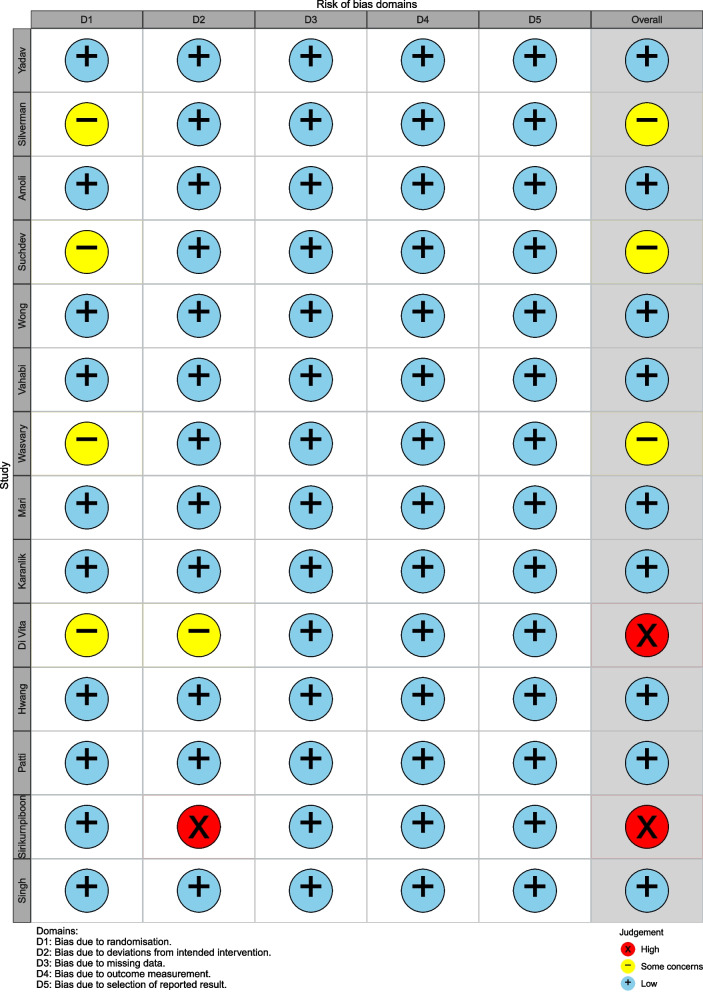
The risk of bias assessment

Figure 2 shows the detailed results of risk of bias. 9 studies had low risk of bias, 3 studies had some concerns of bias risk and 2 studies had high of bias risk. The risk of bias occurring during the randomization process had some concerns in 4 studies [16, 18, 21, 29] due to an uncertain randomization sequence, 1 study [24] did not mention blinding methods and 1 study [27] was categorized as having a high risk of measurement of the outcome because therapists knew the group they were treating.

### Meta-analysis

**Fig. 3:**
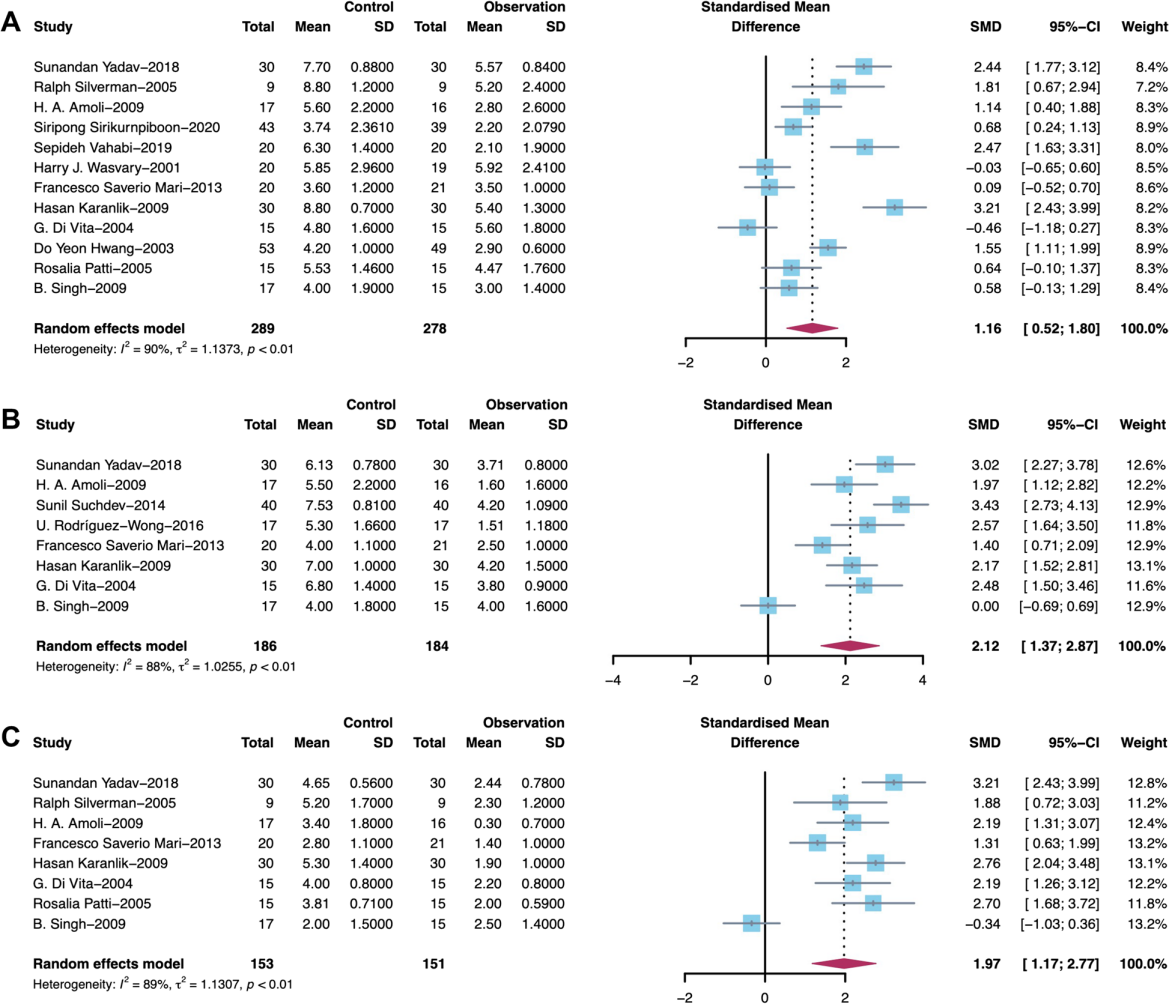
Forest plot based on VAS score on Days 1, 2 and 7 after surgery

Postoperative pain was assessed in 14 studies through a 10-point VAS (0 = no pain, 10 = severe pain). The measured outcomes on days 1, 2 and 7 after the operation were compared because of the variation in the pain assessment time among the studies. The pooled standardized mean difference (SMD) in the degree of pain score was 1.16 (95% CI 0.52 to 1.80, I^2^ = 90%) on day 1 (Fig. 3A), 2.12 (95% CI 1.37 to 2.87, I^2^ = 88%) on day 2 (Fig. 3B), and 1.97 (95% CI 1.17 to 2.77, I^2^ = 89%) on day 7 (Fig. 3C) after the operation.

### Subgroup meta-analysis

**Fig. 4:**
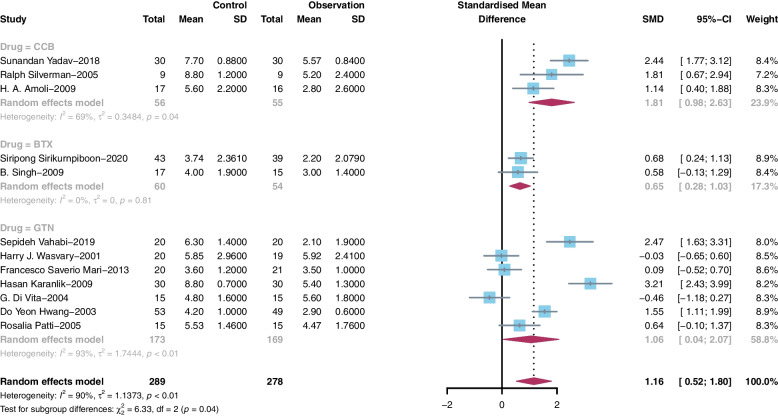
Subgroup meta-analysis on Day 1 after surgery

**Fig. 5:**
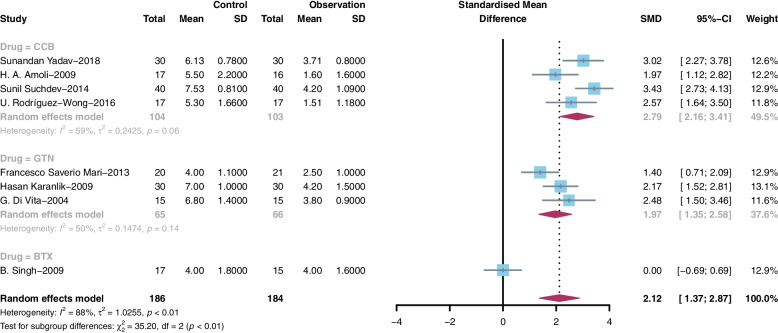
Subgroup meta-analysis on Day 2 after surgery

**Fig. 6:**
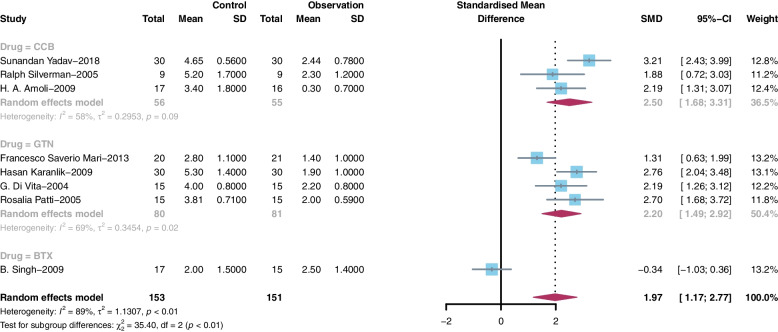
Subgroup meta-analysis on Day 7 after surgery

We performed subgroup analysis according to the kind of drug use. On day 1, the patients treated with CCB (SMD = 1.81, 95% CI 0.98 to 2.63, I^2^ = 69%), BTX (SMD = 0.65, 95% CI 0.28 to 1.03, I^2^ = 0%) or GTN (SMD = 1.06, 95% CI 0.04 to 2.07, I^2^ = 93%) showed lower pain scores than each control group, and CCB showed better pain relief function than the other two drugs (Fig. 4). The same results were shown on day 2 (Fig. 5) and day 7 (Fig. 6). On day 2, the patients treated with CCB (SMD = 2.79, 95% CI 2.16 to 3.41, I^2^ = 59%) showed lower pain scores than those treated with GTN (SMD = 1.97, 95% CI 1.35 to 2.58, I^2^ = 50%), and the patients treated with BTX (SMD = 0, 95% CI -0.69 to 0.69) had no significant difference in pain scores compared with those who were not treated with BTX. On day 7, the patients treated with CCB (SMD = 2.09, 95% CI 1.52 to 2.65, I^2^ = 15%) showed lower pain scores than the patients in the control group, while those treated with GTN (SMD = 1.21, 95% CI -0.20 to 2.62, I^2^ = 92%) and BTX (SMD = -0.66, 95% CI -1.37 to 0.06) had no significant difference in pain scores compared with those who were not treated with these medications.

### Publication bias

We detected publication bias based on Egger’s test in this study. As shown in Table 2, every p value of day 1, day 2 and day 7 was larger than 0.05, which means that there was no significant publication bias in our meta-analysis.

**Table 2:** Publication bias of meta-analysis

	**Egger’s text**	***p*** **value**
Day1	2.467	0.526
Day2	4.529	0.587
Day7	5.651	0.398

## Discussion

Haemorrhoids are a common disease that causes patient inconvenience in life and work and are divided into internal and external haemorrhoids. Mixed haemorrhoids are a mixture of internal and external haemorrhoids, and the most common symptoms of mixed haemorrhoids are bleeding, prolapse, perianal itching, pain and sometimes anaemia secondary to haemorrhage [30]. For mixed haemorrhoids, the treatment options include conservative treatment and surgical treatment. Surgery is the initial treatment of choice in patients with symptomatic grade III–IV haemorrhoids [31]; however, recovering from haemorrhoid surgery is difficult for most patients. This is because after surgery, especially excisional haemorrhoidectomy, postoperative pain, which generally results from a spasm of the internal anal sphincter, causes many issues for patients [2]. For the treatment of postoperative pain in patients after haemorrhoidectomy, people often choose different methods, such as sitz baths [29], medications to reduce swelling [32] or topical anaesthesia [33]. Chemical sphincterotomy, which is always used for patients with anal fissures, can reduce spasms of the internal anal sphincter and relieve pain [34]. Compared with lateral internal sphincterotomy, chemical sphincterotomy, although slightly inferior in analgesic effect, is more advantageous in regard to complications such as faecal incontinence [35]. The most common agents used for chemical sphincterotomy are CCB, GTN and BTX, which have different mechanisms to achieve the desired effects. CCB can reduce myocyte uptake of calcium ions, thus decreasing sphincter contraction or spasm [3]. GTN is a nitric oxide donor and thus aids in the relaxation of the internal sphincter. GTN may also increase blood flow and help in the healing process [36]. BTX, which is produced by the Clostridium botulinum anaerobic bacterium, functions by preventing the secretion of acetylcholine that causes neuromuscular blockage and muscle paralysis [37]. Because of the efficacy of chemical sphincterotomy in anal fissures, doctors pay attention and treat posthemorrhoidectomy pain by using chemical sphincterotomy, and it has been indicated that chemical sphincterotomy also has a good effect after haemorrhoid surgery in pain relief [5].

Our meta-analysis assessed whether chemical sphincterotomy can relieve posthemorrhoidectomy pain. This study included 681 participants from 14 cohort studies and had no significant publication bias based on the results of Egger’s test (all p > 0.05). In our study, we proved that on days 1, 2, and 7 after surgery, the patients treated with chemical sphincterotomy had lower VAS score than those treated with placebo. The difference between these two groups was significant; however, the studies included in our analysis displayed considerable heterogeneity, which may be because of the different surgical approaches and different kinds of experimental drugs. In our selected studies, the surgical approaches included Milligan-Morgan, Ferguson, and stapled haemorrhoidopexy, and different kinds of surgery led to different degrees of pain [2], thus resulting in high heterogeneity. On the other hand, CCB, GTN and BTX also have different effectiveness for pain relief [38]. To compare the differences between these three drugs, we conducted a subgroup meta-analysis. In the subgroup meta-analysis, the heterogeneity decreased in each subgroup, and we found that on days 1, 2 and 7 after surgery, CCB showed better pain relief function than GTN and BTX, indicating that CCB may be a better drug to relieve posthemorrhoidectomy pain caused by spasms of the internal anal sphincter. In an RCT for children who suffered from anal fissures, CCB was more effective and safer than GTN and lidocaine [39], which is consistent with our results above. BTX is injected once into the intersphincteric region of patients immediately after excision [27]. Patients will not receive BTX injection later, and the duration of BTX function may not last long, which may be the reason that BTX only showed pain relief function on Day 1 in our subgroup analysis.

Chemical sphincterotomy also has some shortcomings, such as headache and other side effects, especially when using GTN. Among the studies we included, 4 studies recorded that patients had headaches after using GTN [22, 23, 25, 26], but the headaches could be relieved by dose reduction, which could relieve the headache effectively while having little influence on spasm relief [22] or could be relieved by some medications, such as anti-inflammatory drugs (NAISDs) [23] or prednisolone [40]. Chemical sphincterotomy will also improve the risk of incontinence compared with the use of placebo, but compared with internal sphincterotomy, the incontinence caused by chemical sphincterotomy is less [3]. Moreover, the incontinence is reversible, which means that patients can stop suffering from it when they stop the drug treatment, which is a safer alternative.

There are also several limitations in our study. First, as we have mentioned above, the surgical techniques and the experimental drug dosage application differed across all studies, which resulted in high heterogeneity. Although we conducted a subgroup meta-analysis based on the kind of experimental drug and partly decreased the heterogeneity, in some subgroups, the heterogeneity was still high. Second, the sample size of some trials was small. Silverman’s study [16] only included 9 patients in each observation group and control group, and further research should be conducted, especially studies with a large number of research bases and well-designed RCTs for specific patients.

In conclusion, our study revealed that chemical sphincterotomy application after haemorrhoidectomy significantly decreases pain compared with a placebo. In the comparison of the three experimental drugs, CCB showed better pain relief function than GTN and BTX. As a result, patients can be given CCB ointment after haemorrhoidectomy to help relieve pain.

## Data Availability

The data that support the findings of this study are available from the first author (Yifan Cheng) upon reasonable request.
